# Fast-Track Programs in Total Hip and Knee Replacement at Swedish Hospitals—Influence on 2-Year Risk of Revision and Mortality

**DOI:** 10.3390/jcm10081680

**Published:** 2021-04-14

**Authors:** Urban Berg, Annette W-Dahl, Anna Nilsdotter, Emma Nauclér, Martin Sundberg, Ola Rolfson

**Affiliations:** 1 Department of Orthopaedics, Institute of Clinical Sciences, Sahlgrenska Academy, University of Gothenburg, Göteborgsvägen 31, 431 80 Mölndal, Sweden; anna.nilsdotter@vgregion.se (A.N.); ola.rolfson@vgregion.se (O.R.); 2 Swedish Hip Arthroplasty Register, Registercentrum Västra Götaland, 413 45 Gothenburg, Sweden; emma.naucler@vgregion.se; 3 Orthopedics, Department of Clinical Sciences, Lund University, BMC F12, 221 84 Lund, Sweden; annette.w-dahl@med.lu.se (A.W.-D.); martin.sundberg@med.lu.se (M.S.); 4 The Swedish Knee Arthroplasty Register, Lund University Hospital, 221 85 Lund, Sweden

**Keywords:** fast-track, total hip replacement, total knee replacement, risk of revision, mortality

## Abstract

Purpose: We aimed to study the influence of fast-track care programs in total hip and total knee replacements (THR and TKR) at Swedish hospitals on the risk of revision and mortality within 2 years after the operation. Methods: Data were collected from the Swedish Hip and Knee Arthroplasty Registers (SHAR and SKAR), including 67,913 THR and 59,268 TKR operations from 2011 to 2015 on patients with osteoarthritis. Operations from 2011 to 2015 Revision and mortality in the fast-track group were compared with non-fast-track using Kaplan–Meier survival analysis and Cox regression analysis with adjustments. Results: The hazard ratio (HR) for revision within 2 years after THR with fast-track was 1.19 (CI: 1.03–1.39), indicating increased risk, whereas no increased risk was found in TKR (HR 0.91; CI: 0.79–1.06). The risk of death within 2 years was estimated with a HR of 0.85 (CI: 0.74–0.97) for TKR and 0.96 (CI: 0.85–1.09) for THR in fast-track hospitals compared to non-fast-track. Conclusions: Fast-track programs at Swedish hospitals were associated with an increased risk of revision in THR but not in TKR, while we found the mortality to be lower (TKR) or similar (THR) as compared to non-fast track.

## 1. Introduction

The primary goal of fast-track care programs is to enhance and accelerate recovery after surgery whilst maintaining patient safety [1]. Some publications have reported an increased risk of revision associated with the implementation of a fast-track program in total hip and knee replacement (THR/TKR) [2,3], but it has been assumed that other reasons than the care program may explain the higher revision rate. Other studies have not confirmed an increased risk of revision associated to fast-track in THR and TKR [4,5]. Some uncertainty remains as the control groups have mainly been historical cohorts and the follow-up periods of various length.

The influence of fast-track programs on mortality has also been an issue. It has been concluded that THR is associated with an increased risk of death within 30 days in patients without co-morbidity and age under 60 years due to risks related to surgical intervention, but the long-term mortality is lower in patients after THR compared to a matched population [6]. According to studies from United Kingdom (UK), the mortality rate has been lower within 90 days [7] and 2 years [8] after introduction of fast-track programs in THR and TKR compared to mortality in conventional care programs, but regression analysis with adjustments for confounding factors are lacking.

During the period 2011–2015, the fast-track care concept was implemented at most Swedish hospitals performing THR and TKR [9]. Fast-track programs were defined by the the logistical criteria of admission on the day of surgery, mobilization very early within a few hours after the operation, and discharge as soon as criteria of self-depency and pain control were met. In most other aspects, the treatment protocols were similar in hospitals defined as not fast-track. The median hospital stay during the years 2011–2015 was 3 days in hospitals that had introduced fast-track and 5 days in other hospitals [10]. We aimed to study the influence on revision risk within 2 years after surgery and the mortality within 30 days, 90 days, and 2 years after surgery by analyzing data from the Swedish Hip and Knee Arthroplasty Registers (SHAR and SKAR). 

## 2. Material and Methods

### 2.1. Ethics

The study was in accordance with regulations and relevant guidelines with ethical approval by the Regional Ethical Review Board in Gothenburg, Sweden: Dnr: 2019-00559/1095-18; Exp. 2019-01-10. 

### 2.2. Informed Consent

The need of informed consent for this study was waived by the Regional Ethical Review Board in Gothenburg. 

### 2.3. Definition of Cohorts

The study was based on a survey of the care programs in elective THR and TKR at Swedish hospitals 2011–2015 [9]. Information of the care program was received from 63 hospitals out of 83 performing THR and 63 of 77 performing TKR during this period. In the beginning of the survey period, about 30% of the hospitals had already introduced fast-track but at the end of 2015 fast-track had been implemented at almost 80% of the hospitals performing elective THR and TKR. The operations at hospitals with a known care process were divided into two groups of THR and two groups of TKR, respectively, depending on if the operations were made after implementation of a fast-track program or not. The definition of fast-track was based on the following criteria: (1) admission on the day of surgery, (2) mobilization within 3–6 h after operation, and (3) functional discharge criteria in practice [11]. According to a survey exploring the care programs of elective joint replacements at Swedish hospitals [9], all hospitals had preoperative oral and written information, spinal anesthesia without opioids was the method of choice for anesthesia, and all hospitals had adopted a regime of multimodal analgesia with some variations of drugs that were used. The main difference between the hospitals that had introduced fast-track and those that had not was the logistic criteria and a shorter length of hospital stay as a consequence of the applied principles. A third cohort consisted of operations at hospitals not responding to the questionnaire about the clinical pathway and care program. This cohort had consequently an unknown care program. In the cohorts of THR and TKR with unknown care program, there were more low-volume and private hospitals.

### 2.4. Data Source and Patient Selection

**Figure 1:**
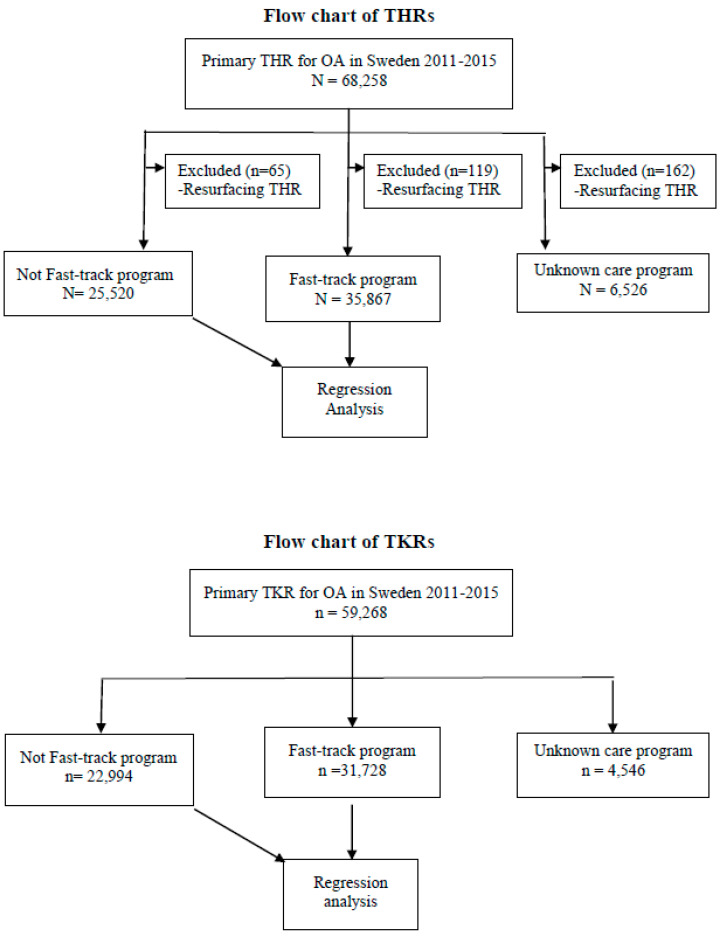
Flow chart of the study. (THR = Total Hip Replacement; TKR = Total Knee Replacement; OA = Osteoarthritis; n = Number of operations).

Data were collected from the SHAR and SKAR and included THR operations (Nordic Medico-Statistical Committee classification of surgical procedures codes NFB29, NFB39, NFB49, and NFB62) and TKR operations (NGB29, NGB39, and NGB49) in patients with osteoarthritis in the hip (International Classification of Diseases 10th revision codes M16.0–M16.9) and the knee (M17.0–M17.5) during the period 2011–2015 (Figure 1). The registers have a national coverage of 100% and the completeness is 96–98% for primary THRs and TKRs. The completeness of revisions is > 90% [12,13]. The data included demographic and procedure-specific variables, date, and main reason for revision and date of death if it occurred within 2 years after the primary joint replacement. Information on deaths is included in the arthroplasty register data by linkage from the Swedish Tax Agency.

### 2.5. Exposure and Outcome

We investigated the exposure of different care programs (fast-track/non-fast-track) in practice at the time of operation (THR/TKR). The outcomes were revision within 2 years and mortality within 30 days, 90 days, and 2 years after surgery. Revision was defined as a re-operation in which one or more of the implanted components were exchanged, removed, or added.

### 2.6. Statistics and Data Analysis 

The Kaplan–Meier method was used to analyze deaths and revisions within 2 years in the fast-track and non-fast-track programs. Furthermore, Cox regression models were used to investigate the risk of revisions within 2 years and mortality within 30 days, 90 days, and 2 years, in the two cohorts. Hazard ratios (HR) together with 95% confidence intervals (CI) are provided. Univariable and multivariable models were explored. In the multivariable model of THR, adjustments were made for age, sex, body mass index (BMI), American Society of Anesthesiologists Physical Status Classification System (ASA class), type of fixation, surgical approach, and year of operation. For TKR, the following factors were assumed to have an impact on mortality: age, sex, BMI, ASA class, and year of operation. In the analysis of revision risk for TKRs, adjustments were also made for the type of fixation (cemented or not cemented) and TKR with or without patella resurfacing. 

## 3. Results

### 3.1. Cohort Characteristics and Revisions after THR

Overall, the demographics and procedure-specific variables were similar in the three cohorts (Table 1). However, some differences may be pointed out, as slightly more males and operations of patients were classified as ASA I in the fast-track cohort. In the fast-track group, direct lateral incision and totally or partially uncemented fixations were more frequent than in the non-fast-track group. The resurfacing THRs were excluded from the comparing analysis. 

The proportion of THRs with a revision within 2 years was 1.3–1.6% respectively in the two cohorts (Table 2). Prosthetic Joint Infection (PJI) or suspected PJI was the most common reason for revision. 

**Figure 2:**
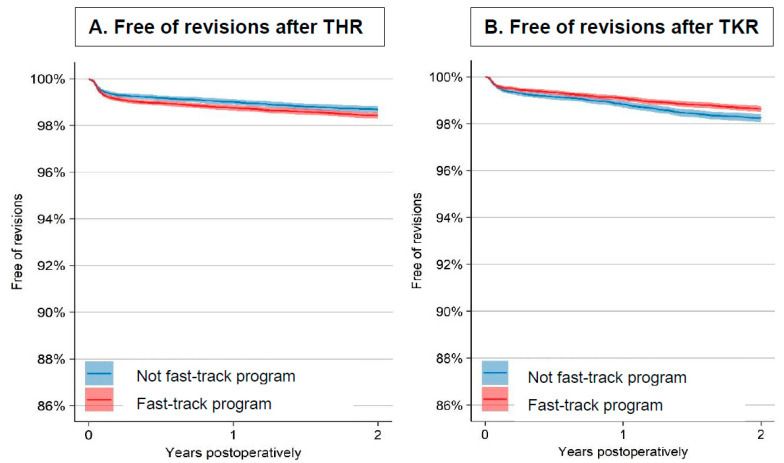
Kaplan–Meier curves for implant survival (free of revisions) within 2 years after THR and TKR. Y-axis truncated in the diagram. (**A**) Free of revisions after THR; (**B**) Free of revisions after TKR.

The 2-year implant survival probability in the fast-track group was 98.4% (95% CI: 98.3, 98.5) and 98.7% (95% CI: 98.5, 98.8) in the non-fast-track group (Figure 2A).

**Figure 3:**
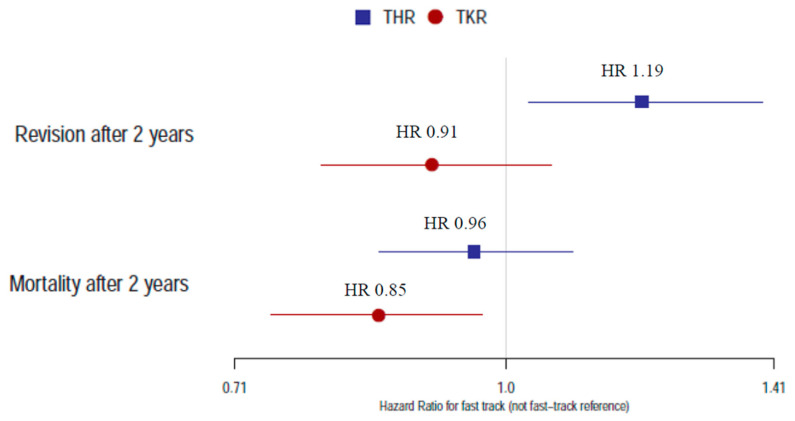
Hazard ratio (95% CI) for revisions and mortality within 2 years in fast-track programs of THR and TKR with non-fast-track as reference. HR: hazard ratio; CI: confidence interval.

The Cox regression analysis with adjustments indicated that the risk of revision within 2 years after the primary THR was approximately 20% higher with fast-track programs compared to not fast-track as the HR was 1.19 (95 CI: 1.03, 1.39) (Figure 3). However, the type of fixation and ASA class were stronger predictors for revision than the care program (Supplementary data, Table S1). 

### 3.2. Cohort Characteristics and Revisions after TKR

The demographics were similar in the cohorts of TKR (Table 1). The revision rate within 2 years was 1.3% in the fast-track cohort compared to 1.6% in the non-fast-track cohort, a difference that was not statistically significant after adjustments (Table 2). The most common reason for revision was PJI with a slightly lower proportion in the fast-track group. The probability of implant survival after 2 years was 98.6% (95% CI: 98.5, 98.8) in the fast-track group and 98.2% (95% CI: 98.1, 98.4) in the non-fast-track group (Figure 2B).

The Cox regression analysis indicated that the risk of revision within 2 years was similar in the fast-track and the non-fast-track program with HR 0.91 (95% CI: 0.79, 1.06) (Figure 3). 

### 3.3. Mortality 

**Figure 4:**
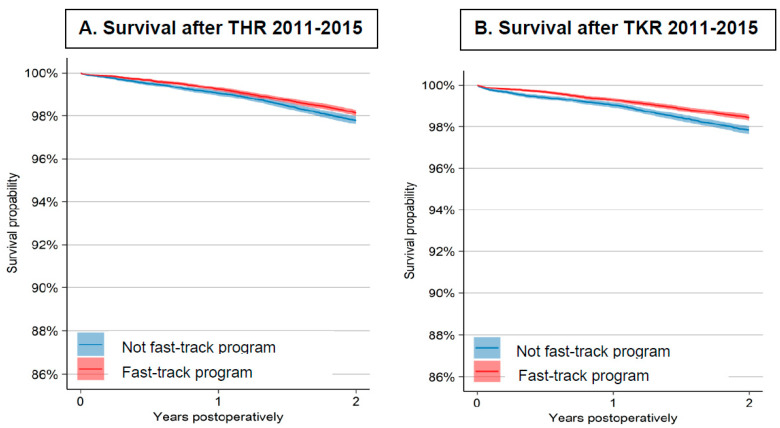
Kaplan–Meier curves of survival within 2 years after THR and TKR. Y-axis truncated in the diagram. (**A**) Survival after THR 2011–2015; (**B**) Survival after TKR 2011–2015.

The 2-year survival probability after THR according to the Kaplan–Meier analysis was 98.1% (95% CI: 98.0, 98.3) in the fast-track group and 97.8% (95% CI: 97.6, 98.0) in the non-fast-track group. In TKR, the estimates of 2-year survival were 98.4% (95% CI: 98.3, 98.6) with fast-track and 97.8% (95% CI: 97.7, 98.0) with the non-fast-track program (Figure 4). 

The Cox regression analysis indicated similar mortality risk after THR with fast-track since the HR was 0.96 (95% CI: 0.85, 1.09) after adjustments. Mortality risk within 2 years after TKR was lower in the fast-track group, HR 0.85 (95% CI: 0.74, 0.97) (Figure 3). 

For both THA and TKR, ASA class was shown to have the strongest association with mortality within 2 years (supplementary data, Table S2). The HRs of mortality and revision within 2 years for the fast-track program are visualized in the forest plot (Figure 3). The mortality within 30 and 90 days was lower in the fast-track group for both THR and TKR, and the estimated HR of death within 90 days was 0.80 (95% CI: 0.55, 1.17) after THR and 0.69 (95% CI: 0.45, 1.07) after TKR (supplementary data, Tables S3 and S4).

## 4. Discussion

The revision rate within 2 years was low in both THR and TKR, but the increased risk of revision after THR in fast-track hospitals raises concerns. Even after adjustments for the strong confounding factors as ASA class, age, and fixation method, there was an increased risk of approximately 20% associated with the fast-track programs. The dominating cause of revision was PJI in all cohorts. Experiences from implementation of fast-track in other countries have pointed out a risk of increased infection and revision rate in the early period for THRs [2,3]. In Denmark, there was an increase of readmissions within 90 days due to infections during the first years after introduction of fast-track in THR and TKR, but later the infection-related readmissions declined and the increased risk could no longer be confirmed [5].

According to the survey of care programs at Swedish hospitals preceding the present study [9], the antibiotic regime did not differ between the care programs. Tranexamic acid was used at all hospitals whereas high-dose systemic glucocorticoids in the preoperative medication were not used routinely in any of the care programs. There were considerable similarities between the fast-track and non-fast-track care programs. 

One basic idea in the fast-track philosophy is “first better—then faster” [14]. However, implementation of a new care program with new routines and very short hospital stay is often associated with a demand for increased efficiency and higher tempo, which may be a challenge. In the Netherlands an increased risk of surgical site infections (SSI) after THR and TKR has also been reported that might be associated with the broad-based implementation of fast-track [15]. The authors suggested that the early mobilization may result in suture dehiscence and increased wound leakage, which may consequently increase the risk of SSI. In addition, falls during the first week after THR and TKR are quite common [16], and the early discharge may be a risk factor if wound leakage is provoked by avoidable falls. However, we have not found any increased risk of infection after TKR in the fast-track programs, and the causes of the increased risk of infection in THR remain unclear.

Our results indicate that fast-track programs may be at least as safe as conventional care regarding mortality within 2 years. In TKR, the mortality rate was shown to be even lower with fast-track but the difference was small. The difference in favor of fast-track may be even larger regarding 90-day mortality, but the low number of fatal events within 90 days necessitates large cohorts to confirm a difference that may exist. A trend of declining short-term mortality rate after THR and TKR during the last decades has been reported despite an increased presurgical comorbidity [17]. 

The implementation of fast-track in THR and TKR has been associated with an additional decrease in short- and medium-term mortality rate [8,18], but adjustments for confounding factors are lacking in previous studies. According to studies from Denmark, the incidences of myocardial infarction (MI) [19] and thromboembolic events (TEE) [20] were lower in fast-track settings compared to a large nationwide retrospective cohort study exploring the risk of cardiovascular events and deaths after THR and TKR [21]. The concept of fast-track, which aims to reduce surgical stress and minimize organ dysfunction, may contribute to the decreased mortality, but about one third of the deaths during the first 90-postoperative days were not considered to be related to surgery [22].

### Strengths and Limitations

The study explores the association between a nationwide implementation of fast-track and the risk of revision and mortality after THR and TKR in Sweden with a high completeness and quality of register data for revision and death. Adjustments for demographic and procedure-specific variables have been made to assess the influence of care programs during the period of broad-based implementation of fast-track. A limitation of the study is that re-operations, not defined as revision, were not included due to inconsequently reporting to the registers. Furthermore, it is a challenge to interpret and compare the results as fast-track is defined by the care principles and variations may occur between different contexts how the care principles are applied. Another limitation is that the care process is complex, and potentially confounding factors not included in our study may influence the outcome. We have to be careful about claiming an existing causal influence of the care program.

## 5. Conclusions

The low mortality within 2 years after both THR and TKR indicate that the procedures are safe independently of care program. The fast-track program had even a lower risk of death within 2 years after TKR, whereas no difference was seen after THR compared to the non-fast-track program. However, implementation of fast-track programs in elective THR and TKR at Swedish hospitals was associated with an increased risk of revision within 2 years in THR but not in TKR. The reason of revision within 2 years was mainly infection, and the cause may be multifactorial, but the demonstrated increased risk of revision necessitates attention to potential weaknesses in the clinical pathway and perioperative routines. 

## Figures and Tables

**Table 1:** Demographic and surgical data on THR and TKR operations 2011–2015.

Variable	Definition	Non-Fast-Track	Fast-Track
THR	n	25,585	35,986
Age (years)	mean (SD)	68.6 (10.4)	68.2 (10.2)
Female sex	n (%)	14,761 (57.7)	20,250 (56.3)
BMI	mean (SD)	27.6 (4.6)	27.3 (4.4)
ASA class	I-II (%)	20,779 (82.7)	29,989 (85.3)
	III-IV (%)	4341 (17.3)	5168 (14.7)
Fixation	Cemented (%)	17,825 (69.7)	22,154 (61.6)
	Hybrid (%)	408 (1.6)	1243 (3.5)
	Uncemented (%)	4404 (17.2)	7123 (19.8)
	Reverse hybrid (%)	2882 (11.3)	5326 (14.8)
Incision	Direct lateral (%)	9187 (35.9)	19,403 (53.9)
	Posterior (%)	15,995 (62.5)	16,573 (46.1)
	Other (%)	402 (1.6)	5 (0.0)
TKR	n	23,036	31,686
Age (years)	mean (SD)	69.6 (9.1)	68.7 (8.9)
Female gender	n (%)	13,372 (58.0)	17,910 (56.5)
BMI	mean (SD)	29.3 (4.7)	28.9 (4.5)
ASA class	I-II (%)	18,826 (81.8)	26,735 (84.5)
	III-IV (%)	4174 (18.2)	4890 (15.5)
Fixation	Cemented (%)	22,284 (96.8)	29,568 (93.4)
Patella resurfacing	No resurfacing (%)	22,210 (96.4)	31,106 (98.2)

THR: total hip replacement; TKR: total knee replacement; BMI: body mass index; ASA: American Society of Anesthesiologists; n: number of operations; SD: standard deviation.

**Table 2:** Revisions within 2 years after THR and TKR operated 2011–2015.

Variable	Definition	Non-Fast-Track	Fast-Track
THR	n	25,520	35,867
Revision <2 years	n (%)	335 (1.3)	565 (1.6)
Reason of revision	PJI or suspected PJI, n (%)	174 (0.7)	300 (0.8)
	Aseptic loosening, n (%)	43 (0.2)	74 (0.2)
	Dislocation, n (%)	61 (0.2)	102 (0.3)
	Fracture, n (%)	38 (0.1)	65 (0.2)
	Other reasons, n (%)	19 (0.1)	24 (0.1)
TKR	n	23,036	31,686
Revision < 2 years	n (%)	405 (1.8)	434 (1.4)
Reason of revision	PJI or suspected PJI	217 (0.9)	221 (0.7)
	Aseptic loosening	36 (0.2)	37 (0.1)
	Patellar problems	71 (0.3)	60 (0.2)
	Instability	42 (0.2)	66 (0.2)
	Other reasons	39 (0.2)	49 (0.2)

THR: total hip replacement; TKR: total knee replacement; PJI: prosthetic joint infection; n: number of operations.

## Data Availability

Data is contained within the article or supplementary material.

## Supplementary Materials

The following are available online at https://www.mdpi.com/article/10.3390/jcm10081680/s1, Table S1: Multivariable cox regression analysis of revision for any reason within 2 years after THR and TKR. Table S2: Multivariable cox regression analysis of death within 2 years after THR and TKR. Table S3: Mortality within 30 days, 90 days and 2 years after THR and TKR in different care programs 2011–2015. Table S4: Multivariable cox regression analysis of death within 90 days after THR and TKR.

## References

[1] Wainwright T.W., Kehlet H. (2019). Fast-track hip and knee arthroplasty—have we reached the goal?. Acta Orthop..

[2] Amlie E., Lerdal A., Gay C.L., Høvik Ø., Nordsletten L., Dimmen S. (2016). A Trend for Increased Risk of Revision Surgery due to Deep Infection following Fast-Track Hip Arthroplasty. Adv. Orthop..

[3] Pamilo K.J., Torkki P., Peltola M., Pesola M., Remes V., Paloneva J. (2017). Reduced length of uninterrupted institutional stay after implementing a fast-track protocol for primary total hip replacement. Acta Orthop..

[4] Hartog Y.M.D., Mathijssen N.M.C., Vehmeijer S.B.W. (2013). Reduced length of hospital stay after the introduction of a rapid recovery protocol for primary THA procedures. Acta Orthop..

[5] Glassou E.N., Pedersen A.B., Hansen T.B. (2014). Risk of re-admission, reoperation, and mortality within 90 days of total hip and knee arthroplasty in fast-track departments in Denmark from 2005 to 2011. Acta Orthop..

[6] Pedersen A.B., Baron J.A., Overgaard S., Johnsen S.P. (2011). Short- and long-term mortality following primary total hip replacement for osteoarthritis: A Danish nationwide epidemiological study. J. Bone Joint Surg. Br..

[7] Khan S.K., Malviya A., Muller S.D., Carluke I., Partington P.F., Emmerson K.P., Reed M.R. (2013). Reduced short-term complications and mortality following Enhanced Recovery primary hip and knee arthroplasty: Results from 6000 consecutive procedures. Acta Orthop..

[8] Savaridas T., Serrano-Pedraza I., Khan S.K., Martin K., Malviya A., Reed M.R. (2013). Reduced medium-term mortality following primary total hip and knee arthroplasty with an enhanced recovery program. Acta Orthop..

[9] Berg U. (2020). Fast-Track Programs in Total Hip and Knee Replacement at Swedish Hospitals-Influences on Safety, Outcome and Patients’ Experiences. Ph.D. Thesis.

[10] Kärrhom J., Rogmark C., Naucler E., Nåtman J., Vinblad J., Mohaddes M., Rolfson O. (2020). Swedish Hip Arthroplasty Register Annual Report 2019.

[11] Berg U., Bülow E., Sundberg M., Rolfson O. (2018). No increase in readmissions or adverse events after implementation of fast-track program in total hip and knee replacement at 8 Swedish hospitals: An observational before-and-after study of 14,148 total joint replacements 2011–2015. Acta Orthop..

[12] Kärrhom J., Rogmark C., Naucler E., Vinblad J., Mohaddes M., Rolfson O. (2019). Swedish Hip Arthroplasty Register Annual Report 2018.

[13] Robertsson O., Ranstam J., Sundberg M., W-Dahl A., Lidgren L. (2014). The Swedish knee arthroplasty register: A review. Bone Joint Res..

[14] Vehmeijer S.B.W., Husted H., Kehlet H. (2018). Outpatient total hip and knee arthroplasty. Acta Orthop..

[15] Ho J., Meis J.F., Nabuurs-Franssen M., Voss A. (2015). Hip and knee arthroplasty: Quo vadis?. Antimicrob. Resist. Infect. Control..

[16] Jorgensen C.C., Kehlet H. (2013). Fall-related admissions after fast-track total hip and knee arthroplasty—Cause of concern or consequence of success?. Clin. Interv. Aging.

[17] Lalmohamed A., Vestergaard P., de Boer A., Leufkens H.G., van Staa T.P., de Vries F. (2014). Changes in mortality patterns following total hip or knee arthroplasty over the past two decades: A nationwide cohort study. Arthritis Rheumatol..

[18] Deng Q.-F., Gu H.-Y., Peng W.-Y., Zhang Q., Huang Z.-D., Zhang C., Yu Y.-X. (2018). Impact of enhanced recovery after surgery on postoperative recovery after joint arthroplasty: Results from a systematic review and meta-analysis. Postgrad. Med. J..

[19] Petersen P.B., Kehlet H., Jørgensen C.C. (2018). On behalf of the Lundbeck Foundation Center for Fast-track Hip and Knee Replacement Collaborative Group Myocardial infarction following fast-track total hip and knee arthroplasty—incidence, time course, and risk factors: A prospective cohort study of 24,862 procedures. Acta Orthop..

[20] Jorgensen C.C., Kehlet H. (2016). Early thromboembolic events ≤ 1week after fast-track total hip and knee arthroplasty. Thromb. Res..

[21] Pedersen A.B., Mehnert F., Sorensen H.T., Emmeluth C., Overgaard S., Johnsen S.P. (2014). The risk of venous thromboembolism, myocardial infarction, stroke, major bleeding and death in patients undergoing total hip and knee replacement: A 15-year retrospective cohort study of routine clinical practice. Bone Joint J..

[22] Jørgensen C., Kehlet H. (2017). On behalf of the Lundbeck Foundation Centre for Fast-track Hip and Knee Replacement Collaborative Group Time course and reasons for 90-day mortality in fast-track hip and knee arthroplasty. Acta Anaesthesiol. Scand..

