# Supplementary material for: Fast-Track Programs in Total Hip and Knee Replacement at Swedish Hospitals—Influence on 2-Year Risk of Revision and Mortality
Source: J Clin Med. 2021 Apr 14;10(8):1680. doi: 10.3390/jcm10081680 (PMC8070704; doi:10.3390/jcm10081680)
Supplement: Supplementary file 1 [file jcm-10-01680-s001.pdf]

Supplementary data Table S1. Multivariable cox regression analysis of revision for any reason within 2 years after THR and TKR (HR with 95% CI)

			**CI 95%**	
**Operation**	**Variable**	**HR**	**Lower**	**Upper**
THR	Fast-track	1.19	1.03	1.39
	Female	0.67	0.59	0.77
	Age	1.02	1.01	1.03
	Year of operation	0.96	0.91	1.01
	BMI	1.04	1.03	1.06
	ASA II	1.45	1.19	1.76
	ASA III-IV	2.29	1.81	2.90
	Incision Posterior	0.96	0.84	1.10
	Incision Other	0.74	0.30	1.80
	Hybrid	1.07	0.66	1.71
	Uncemented	2.72	2.26	3.27
	Reversed hybrid	1.89	1.55	2.32
TKR	Fast-track	0.91	0.79	1.06
	Female	0.73	0.64	0.84
	Age	0.99	0.98	1.00
	Year of operation	0.86	0.81	0.90
	BMI	1.02	1.00	1.03
	ASA II	1.23	1.00	1.51
	ASA III-V	1.96	1.54	2.50
	Uncemented	1.08	0.79	1.47
	No patella resurfacing	0.94	0.62	1.43

For THR the non-fast-track care program, male sex, ASA I, direct lateral incision and cemented fixation were used as references. For TKR the care program non-fast-track, male sex, ASA I, cemented fixation and TKR without patellar resurfacing were used as references. THR: Total Hip Replacement; TKR: Total Knee Replacement; HR: Hazard Ratio; CI: Confidence Interval; Body Mass Index; ASA: American Society of Anesthesiologists.

Supplementary data Table S2. Multivariable cox regression analysis of death within 2 years after THR and TKR (HR with 95% CI)

			CI 95%	
**Operation**	**Variable**	**HR**	**Lower**	**Upper**
THR	Fast-track	0.96	0.85	1.09
	Female	0.62	0.55	0.69
	Age	1.07	1.06	1.07
	Year of operation	0.95	0.91	0.99
	BMI	0.98	0.97	0.99
	ASA II	2.27	1.77	2.90
	ASA III-IV	5.26	4.07	6.81
	Incision Posterior	0.66	0.25	1.77
	Incision Other	0.95	0.84	1.06
	Hybrid	1.07	0.77	1.48
	Uncemented	0.73	0.57	0.93
	Reversed hybrid	0.67	0.52	0.86
TKR	Fast-track	0.85	0.74	0.97
	Female	0.64	0.56	0.73
	Age	1.08	1.07	1.09
	Year of operation	0.97	0.92	1.02
	BMI	0.99	0.98	1.01
	ASA II	1.76	1.34	2.31
	ASA III-V	3.97	2.98	5.29

For THR the non-fast-track care program, male sex, ASA I, direct lateral incision and cemented fixation were used as references. For TKR the care program non-fast-track, male sex and ASA I was used as references. THR: Total Hip Replacement; TKR: Total Knee Replacement; HR: Hazard Ratio; CI: Confidence Interval; Body Mass Index; ASA: American Society of Anesthesiologists.

Supplementary data Table S3. Mortality within 30 days, 90 days and 2 years after THR and TKR in different care programs 2011-2015

	Variable, Definition	Non-fast-track program	Fast-track program
**THR**	Operations, n	25,520	35,867
	Deaths < 30 days, n (%)	34 (0.1)	37 (0.1)
	Deaths < 90 days, n (%)	66 (0.3)	64 (0.2)
	Deaths < 2 years, n (%)	564 (2.2)	674 (1.9)
**TKR**	Operations, n	23,036	31,686
	Deaths < 30 days, n (%)	25 (0.1)	18 (0.1)
	Deaths < 90 days, n (%)	53 (0.2)	42 (0.1)
	Deaths < 2 years, n (%)	442 (1.9)	453 (1.4)

THR: Total hip replacement, TKR: Total knee replacement, n: number

Supplementary data Table S4. Multivariable cox regression analysis of death within 90 days after THR and TKR (HR with 95% CI)

			CI 95%	
**Operation**	**Variable**	**HR**	**Lower**	**Upper**
THR	Fast-track	0.80	0.55	1.17
	Female	0.58	0.41	0.83
	Age	1.07	1.04	1.10
	Year of operation	0.99	0.87	1.13
	BMI	0.98	0.94	1.02
	ASA II	2.13	0.96	4.75
	ASA III-IV	6.88	3.03	15.6
	Incision Posterior	0.80	0.56	1.15
	Incision Other	1.33	0.18	9.77
	Hybrid	0.54	0.13	2.19
	Uncemented	0.7	0.33	1.51
	Reversed hybrid	0.95	0.48	1.86
TKR	Fast-track	0.69	0.45	1.07
	Female	0.49	0.32	0.74
	Age	1.11	1.08	1.15
	Year of operation	0.95	0.82	1.11
	BMI	1	0.95	1.05
	ASA II	1.68	0.66	4.27
	ASA III-V	4.99	1.92	13.0

For THR the non-fast-track care program, male sex, ASA I, direct lateral incision and cemented fixation were used as references. For TKR the care program non-fast-track, male sex and ASA I was used as references. THR: Total Hip Replacement; TKR: Total Knee Replacement; HR: Hazard Ratio; CI: Confidence Interval; Body Mass Index; ASA: American Society of Anesthesiologists.
